# An atypical presentation of a rare breast tumor

**DOI:** 10.1016/j.jdcr.2026.07.042

**Published:** 2026-07-30

**Authors:** Kenza Khachani, Laila Benzekri, Kaoutar Znati, Nadia Ismaili, Mariame Meziane

**Affiliations:** a Department of Dermatology, Mohammed V University, Ibn Sina University Hospital, Rabat, Morocco; b Department of Pathology, Mohammed V University, Ibn Sina University Hospital, Rabat, Morocco

**Keywords:** adnexal tumor, dermatoscopy, pathology, syringocystadenoma papilliferum

## Case

A 23-year-old woman with no medical history other than previous pregnancy, presented with 3 congenital nodules of the right breast. She was referred for a painful and oozing breast tumor that had rapidly grown over 10 months. This worsening happened during the second trimester of her first pregnancy, significantly impairing her quality of life.

Clinical examination showed a pink, verrucous tumor (2 × 8 × 4 cm), extending in a Blaschko-linear arrangement from the upper quadrants of the right breast to the ipsilateral axillary region (Fig 1).

**Fig 1:**
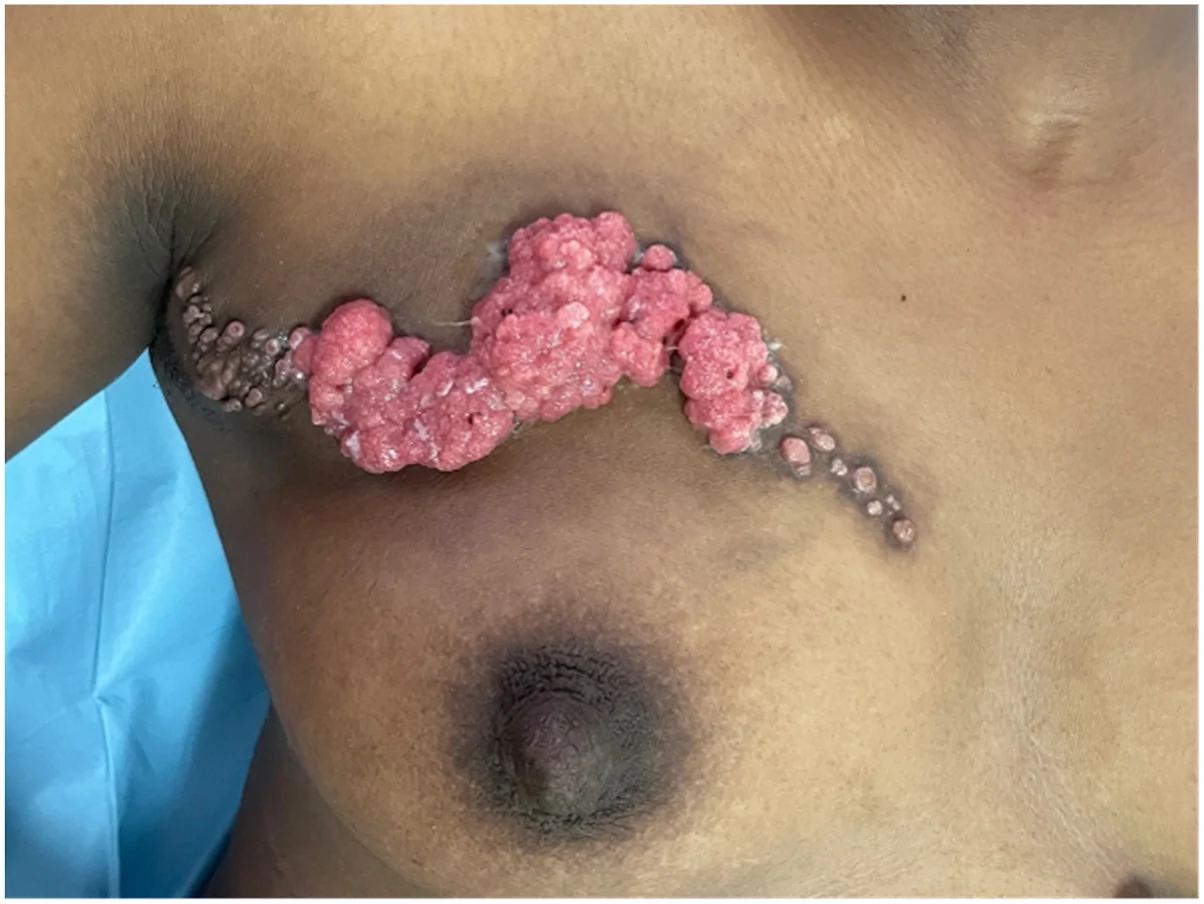
Clinical photograph of the patient’s lesions demonstrating the tumor and the peripheral nodules in a Blaschko-linear arrangement.

Dermatoscopy found an amorphous milky-red background, polymorphic vessels separated by white septa, and a central depression in the nodules (Figs 2 and 3).

**Fig 2:**
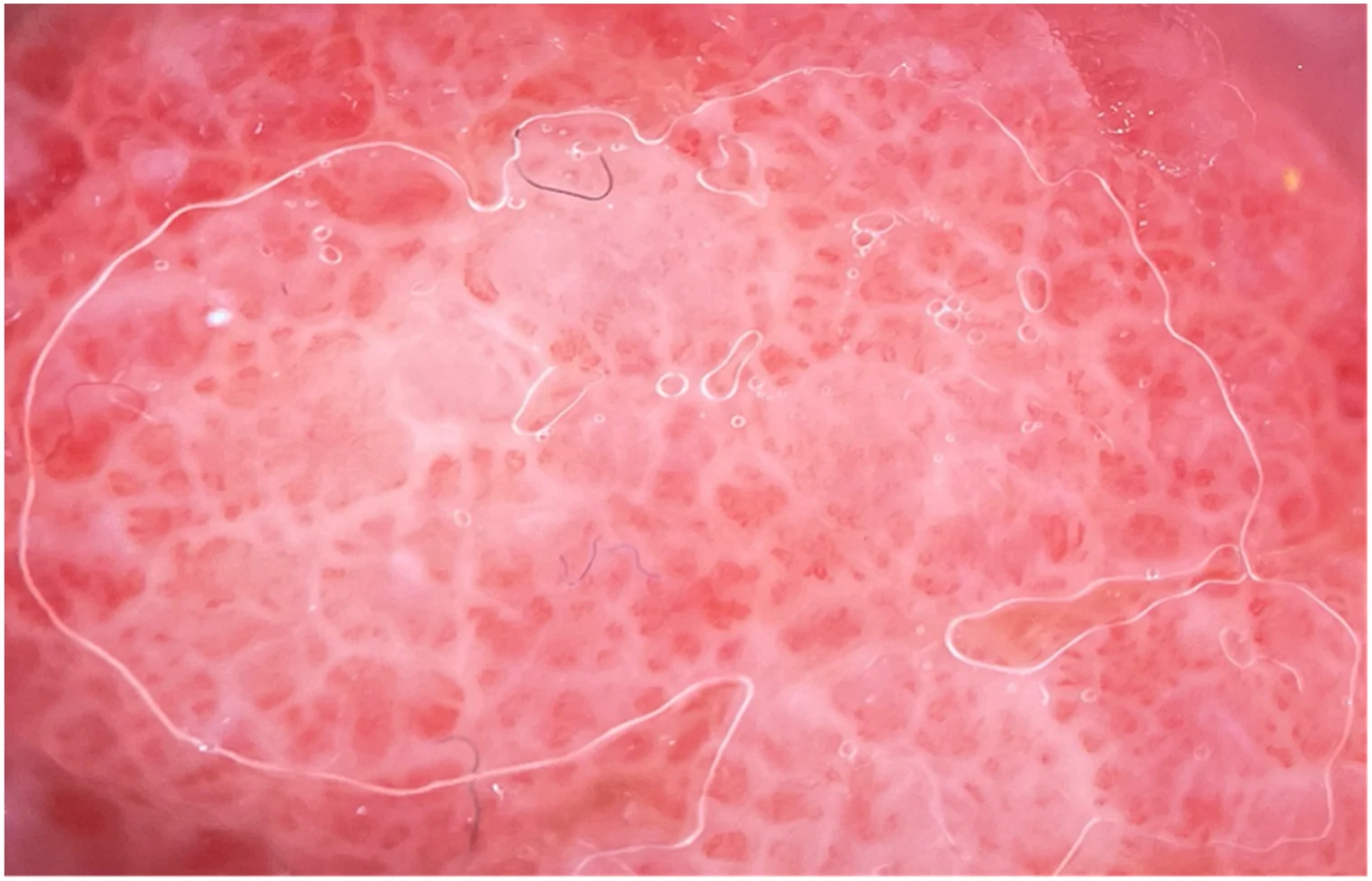
Dermatoscopic aspect of the tumorous part of the syringocystadenoma papilliferum: amorphous milky-red background, irregular and polymorphous vessels separated by white septa, telangiectasias.

**Fig 3:**
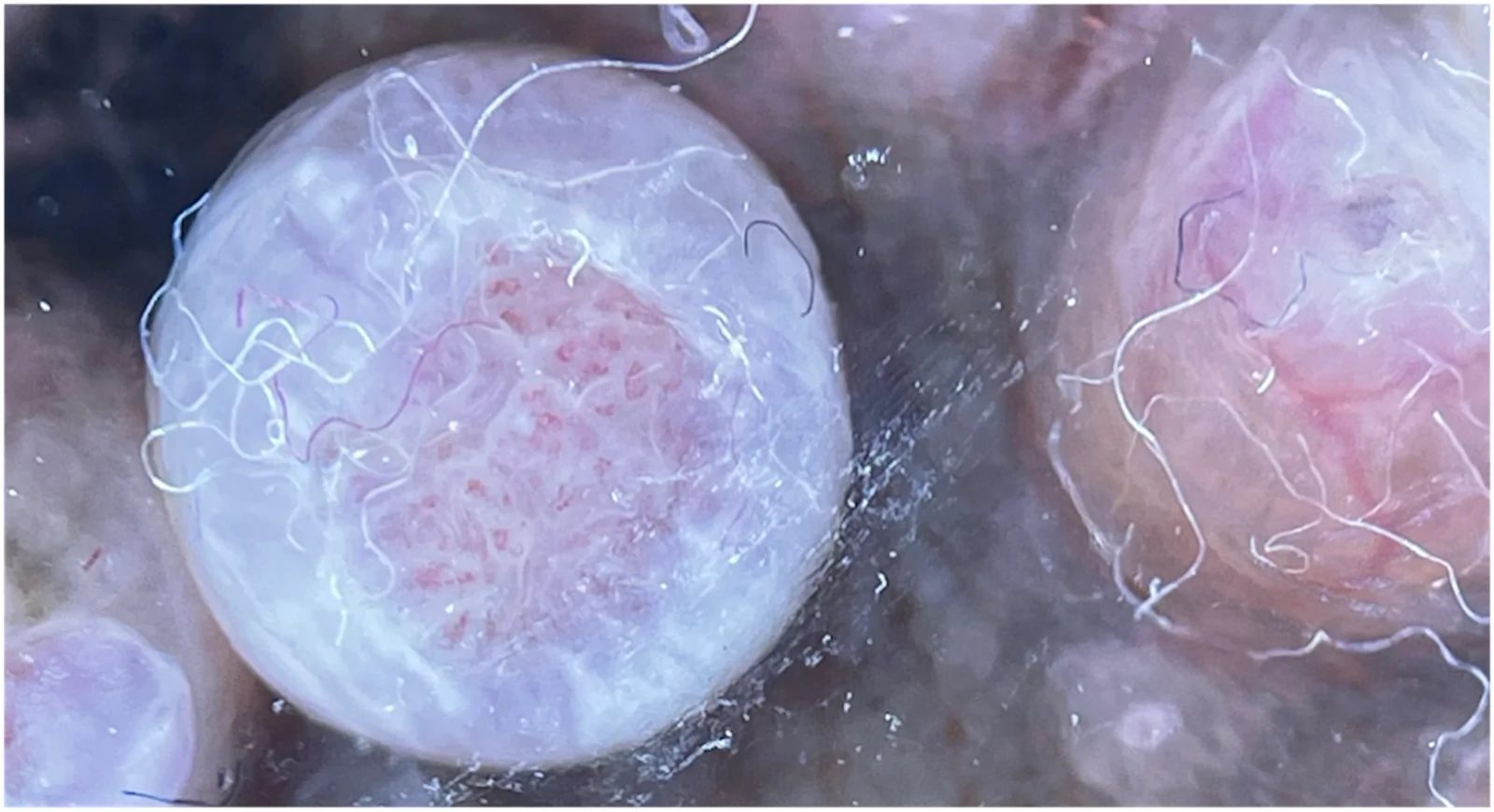
Dermatoscopic aspect of the nodular part of the syringocystadenoma papilliferum: exophytic papillary structures centered by a depression, comprising polymorphic vessels (hairpin, sinuous, arborescent) separated by septa.

Histopathology revealed a papillary architecture made of a double layer of cells, a luminal layer of cylindrical cells, and a basal layer of cuboidal cells, with an inflammatory infiltrate of plasma cells in the papillary stroma (Fig 4).

**Fig 4:**
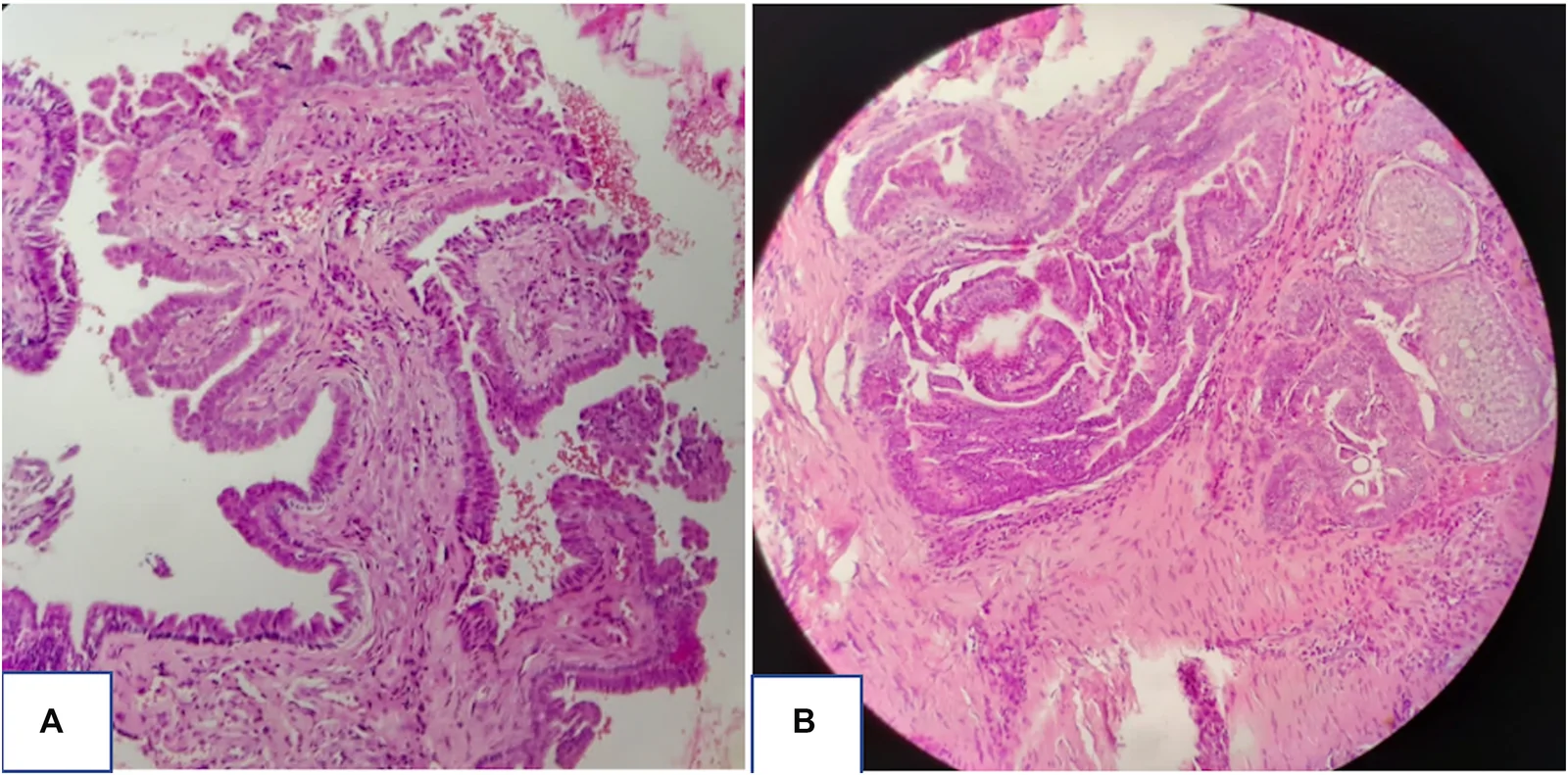
Hematoxylin-eosin stain showing invaginations arising from the cutaneous surface, with a papillary architecture made of double layers: luminal cylindrical cells and basal cubic cells **(A)**. The dermis showed dense interstitial inflammatory infiltrate rich in mature plasma cells **(B)**.

Breast magnetic resonance imaging found a tumor of the upper quadrants extending to the right subscapular chest wall (23 × 78 × 43 mm), with benign ipsilateral axillary adenopathy (Fig 5).

**Fig 5:**
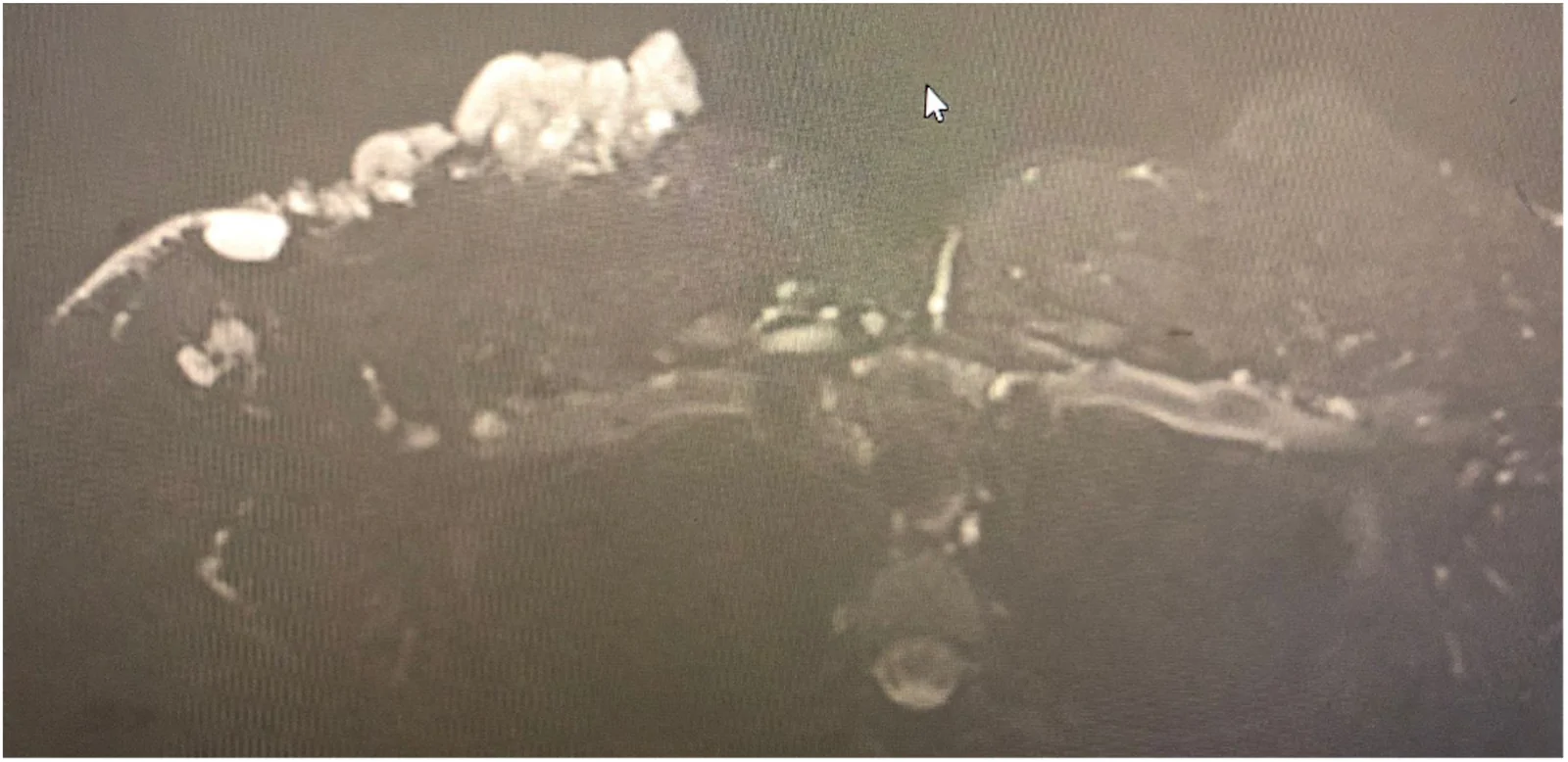
Breast magnetic resonance imaging (gadolinium-enhanced) showing a tumoral mass at the right subscapular chest wall, infiltrating the subcutaneous fat, without involving the glandular tissue or pectoralis muscle.

**Question: What is the most likely diagnosis?**

**A.** Squamous cell carcinoma.

**B.** Porocarcinoma.

**C.** Syringocystadenoma papilliferum.

**D.** Tuberculosis verrucosa cutis.

**E.** Dermatofibrosarcoma protuberans.

## Discussion

Diagnosis: Syringocystadenoma papilliferum (SCAP).

SCAP is a rare, benign adnexal tumor^1^ arising from apocrine glands, either de novo, as seen in our patient, or on a sebaceous hamartoma. It usually involves the head and neck; trunk and acral sites are rarely reported.^2^ Three clinical variants exist: plaque, linear (Blaschko-linear), and nodular. SCAP usually appears at birth or before puberty.^2^ The lesion may enlarge, ulcerate, or become verrucous during puberty or pregnancy,^3^ as in our patient.

Reported dermatoscopic features, also described in our case, include polymorphic vessels, amorphous milky-red and pink areas, central depression, and erosions.^4^ Although dermatoscopy is supportive, the diagnosis relies on histology, showing an exophytic, papillary, duct-like proliferation (cylindrical luminal and cuboidal basal cells) with cystic spaces in a plasma-cell-rich stroma.

The ulcerated, rapidly growing nature of the lesion raised several differential diagnoses. Squamous cell carcinoma and porocarcinoma were excluded because pathology showed no keratinocyte atypia, no dysplastic squamous nests, and no mitoses but showed benign bilayered apocrine proliferation. Dermatofibrosarcoma protuberans was excluded because the lesion showed an epithelial and glandular pattern rather than a storiform spindle-cell proliferation. Tuberculosis verrucosa cutis was ruled out by the absence of caseation necrosis on histology and a negative acid-fast bacilli stain. The Blaschko-linear and verrucous pattern suggested other Blaschko-linear dermatoses (linear epidermal nevus, inflammatory linear verrucous epidermal nevus, and the verrucous stage of incontinentia pigmenti), which were excluded because they typically present in infancy and show only epidermal changes without apocrine papillary architecture or plasma-cell-rich stroma. The dermatoscopic and histologic findings therefore confirmed the diagnosis of SCAP.

Although benign, SCAP can rarely transform into syringocystadenocarcinoma papilliferum.^5^ Syringocystadenocarcinoma papilliferum is typically described as a rapidly growing, papillary or exophytic tumor with ulceration, discharge, or pain. Despite these features in our patient, excision confirmed a benign lesion with clear margins. Reconstruction with a skin graft improved her quality of life.

## Conflicts of interest

None disclosed.
